# Gluten-related nutritional challenges in pediatric subjects: treatment and beyond

**DOI:** 10.3389/fnut.2025.1709121

**Published:** 2025-11-25

**Authors:** Maria Elena Capra, Tullia Sguerso, Valentina Aliverti, Gianlorenzo Pisseri, Arianna Maria Bellani, Martina Berzieri, Anna Giuseppina Montani, Susanna Esposito, Giacomo Biasucci

**Affiliations:** 1 Pediatrics and Neonatology Unit, Guglielmo da Saliceto Hospital, Piacenza, Italy; 2 Pediatric Clinic, Department of Medicine and Surgery, University of Parma, Parma, Italy; 3 Department of Medicine and Surgery, University of Parma, Parma, Italy

**Keywords:** gluten, celiac disease, wheat allergy, non-celiac gluten sensitivity, nutrition, vitamin, challenges

## Abstract

Growing awareness of gluten-related disorders has led to a rising number of diagnoses of celiac disease (CD) and increasing adoption of the gluten-free diet (GFD), often without medical necessity. This narrative review summarizes current evidence on the main gluten-related conditions—CD, wheat allergy (WA), and non-celiac gluten sensitivity (NCGS)—and their nutritional implications, with particular focus on pediatric populations. Although these disorders share overlapping clinical features, they differ in pathogenesis, diagnostic criteria, and management. In CD, strict lifelong gluten exclusion remains essential for intestinal healing and symptom resolution, whereas in WA, wheat avoidance is the cornerstone of therapy. NCGS is characterized by gluten-related gastrointestinal and extra-intestinal symptoms in the absence of CD or WA, with notable clinical overlap with irritable bowel syndrome. Across all conditions, adherence to a GFD can lead to nutritional imbalances, including deficiencies in iron, folate, vitamin B12, vitamin D, calcium, zinc, and magnesium, as well as reduced fiber intake and unfavorable changes in gut microbiota. Overreliance on processed gluten-free foods may further increase cardiometabolic risks. In children, unmonitored GFDs may impair growth and neurodevelopment. Clinicians should ensure accurate differential diagnosis, provide nutritional counseling, and monitor long-term outcomes to balance the therapeutic benefits of GFD with potential risks.

## 1 Background

In recent years, interest in gluten-related disorders has expanded considerably, not only within the medical community but also among the wider public. This heightened awareness has resulted in increased recognition of celiac disease (CD) and, at the same time, a substantial proportion of people choosing to follow a self-initiated gluten-free diet (GFD). Such developments have contributed to widespread uncertainty about the full spectrum of gluten-associated conditions, which encompasses celiac disease, wheat allergy (WA), and non-celiac gluten sensitivity (NCGS). Although these entities share some overlapping clinical features, they are fundamentally distinct with respect to pathophysiology, diagnostic approaches, and therapeutic strategies (1–4).

CD is a chronic, immune-mediated disorder triggered by gluten ingestion in genetically predisposed individuals, leading to characteristic small-bowel mucosal damage and extra-intestinal manifestations. By contrast, WA is an IgE-driven immune reaction that may provoke acute hypersensitivity symptoms following wheat exposure. NCGS refers to a condition in which gluten consumption provokes both gastrointestinal and extra-intestinal complaints, in the absence of autoimmune mechanisms or IgE-mediated allergy (5–8).

Distinguishing among these conditions remains a clinical challenge, particularly in terms of long-term management and therapeutic decision-making. Furthermore, the widespread adoption of GFD has raised concern regarding its nutritional adequacy and possible health risks for individuals without a clear medical indication (9, 10).

The present narrative review seeks to provide a broad overview of gluten-related disorders, emphasizing the key distinctions among CD, WA, and NCGS. In addition, it discusses potential adverse effects associated with GFD and examines emerging evidence on nutritional issues linked to gluten exclusion.

## 2 Methods

This narrative review was conducted in three main stages: an initial literature search, a screening of abstracts and complete manuscripts, and a final evaluation of the selected studies. Publications from 1990 to 2025 were retrieved from major scientific databases, including PubMed, EMBASE, Scopus, ScienceDirect, Web of Science, and Google Scholar, to ensure a comprehensive overview of the available evidence. The most recent search was performed in June 2025.

Eligible study designs comprised randomized placebo-controlled trials, controlled clinical investigations, double-blind randomized studies, and systematic reviews. The search strategy combined the following keywords: “celiac disease” OR “gluten-free diet” OR “wheat allergy” OR “non-celiac gluten sensitivity” AND “diagnosis” OR “treatment” OR “weight changes” OR “cardiovascular disease” OR “dyslipidemia” OR “vitamin” OR “nutritional challenges” OR “eating behavior.” In addition, reference lists of the included publications were manually reviewed to identify further relevant contributions.

Only complete articles published in English were considered. After duplicates were removed, abstracts were screened for relevance, and the remaining manuscripts were assessed against the inclusion criteria. The evidence collected was subsequently synthesized into an integrated narrative review, with a specific focus on nutritional issues related to gluten exposure and restriction.

## 3 Results

### 3.1 Celiac disease

CD is a chronic, immune-mediated disorder of the small intestine, triggered by the ingestion of gluten-containing cereals such as wheat, barley, and rye in genetically predisposed individuals. The condition arises from an abnormal immune reaction to gluten-derived peptides, which induces intestinal inflammation and villous atrophy (11, 12). Both innate and adaptive immune mechanisms are implicated, with HLA-DQ2 and HLA-DQ8 molecules playing a central role by presenting deamidated gluten peptides to CD4+ T cells, thereby initiating the inflammatory cascade (12, 13).

Globally, CD affects roughly 1% of the population, though prevalence rates differ according to geographic, genetic, and environmental factors (14, 15). Recent evidence indicates a rising incidence, likely reflecting increased disease awareness, improved diagnostic tools, and shifts in dietary habits (13, 14, 16). Despite this, many individuals remain undiagnosed, highlighting the importance of enhanced screening strategies (12, 14).

#### 3.1.1 Clinical presentation and diagnosis

The clinical spectrum of CD is highly variable, ranging from classical gastrointestinal symptoms to extra-intestinal features and even silent or asymptomatic forms (Table 1) (12–14). Such heterogeneity frequently results in diagnostic delays, reinforcing the need to consider CD in diverse clinical scenarios (12, 15).

**Table 1:** Celiac disease (CD) clinical manifestations.

**CD clinical manifestations**
- Gastrointestinal symptoms include chronic diarrhea, abdominal pain, bloating, weight loss, failure to thrive in children, and constipation. - Extra-intestinal manifestations may involve short stature/growth delay, iron-deficiency anemia, osteoporosis, dermatitis herpetiformis, neurological symptoms (e.g., peripheral neuropathy, ataxia), and reproductive issues - Associated conditions frequently include type 1 diabetes mellitus, autoimmune thyroid disease, Down syndrome, Turner syndrome, selective IgA deficiency, and first-degree relatives with CD

Rubio-Tapia et al. (12), Lebwohl et al. (13), and Caio et al. (14).

Diagnosis typically requires a combination of serological and, in some cases, histological investigations (11):

- Serological tests: First-line assessments include serum total IgA and IgA anti–tissue transglutaminase antibodies (TGA-IgA). In IgA-deficient individuals, IgG-based assays, such as anti-deamidated gliadin peptide IgG, are recommended (12).
- No-biopsy approach: The 2020 ESPGHAN guidelines allow a diagnosis without biopsy in symptomatic pediatric patients with TGA-IgA ≥10 times the upper limit of normal and positive endomysial antibodies (EMA-IgA) on a second sample.
- Histological evaluation: When serological findings are discordant or below the threshold, duodenal biopsies remain necessary. Current recommendations call for at least four samples from the distal duodenum and one from the bulb (11).
- HLA typing: Although not routinely required, genotyping for HLA-DQ2 and DQ8 can help exclude CD, as the absence of these alleles makes the diagnosis highly improbable (13).

All testing should be performed while the patient consumes a gluten-containing diet, to minimize false negatives and maintain diagnostic reliability (11).

#### 3.1.2 Treatment and follow-up

The only effective therapy for CD is lifelong adherence to a strict gluten-free diet, which generally results in clinical remission and restoration of mucosal integrity (11, 12). Compliance must be monitored through regular clinical evaluations and repeated measurement of TGA-IgA (11). Follow-up visits are typically advised at 3–6 months post-diagnosis, and subsequently every 6–12 months depending on clinical status and serological trends (13).

If symptoms persist or antibody levels remain elevated, dietary adherence should be reassessed, and alternative explanations such as refractory CD or coexisting conditions should be investigated (14, 16).

### 3.2 Wheat allergy

WA is an immune-mediated hypersensitivity reaction that may involve IgE- or non-IgE–dependent mechanisms against wheat proteins such as albumins, globulins, gliadins, and glutenins. Unlike celiac disease, which represents an autoimmune condition, WA is a typical food allergy characterized by an inappropriate immune response directed at wheat-derived antigens (17). The IgE-mediated pathway commonly involves mast cell and basophil activation with subsequent histamine and mediator release (18).

#### 3.2.1 Clinical manifestations and diagnosis

WA can present in several distinct clinical forms, including immediate-type food allergy, wheat-dependent exercise-induced anaphylaxis (WDEIA), occupational asthma or rhinitis (commonly referred to as baker's asthma), and contact urticaria (19).

The symptoms vary depending on the underlying immune mechanism and the route of exposure (Table 2) (17–21). Accurate differentiation between WA, CD, and NCGS is essential, as their pathophysiology, clinical profile, and therapeutic approaches differ significantly (22).

**Table 2:** Wheat-allergy (WA) clinical manifestations.

**WA clinical manifestations**
• IgE-mediated reactions: occur within minutes to hours after wheat ingestion and include urticaria, angioedema, nausea, vomiting, abdominal pain, respiratory symptoms, and potentially anaphylaxis • Non-IgE-mediated reactions: involve delayed gastrointestinal symptoms such as vomiting, diarrhea, and abdominal discomfort, especially in children (e.g., food protein-induced enterocolitis syndrome - FPIES) • WDEIA: occurs when wheat ingestion is followed by physical exertion and can result in life-threatening anaphylaxis • WA is more common in children, with many outgrowing the allergy by adolescence, although persistence into adulthood is possible. Diagnosis of WA relies on a combination of clinical history, *in vivo* and *in vitro* testing: • Skin prick tests (SPT) and serum-specific IgE tests (e.g., to wheat, ω-5 gliadin) are used to identify sensitization • Component-resolved diagnostics (CRD) may improve specificity, especially in WDEIA • Oral food challenge (OFC) remains the gold standard for diagnosis, particularly when test results are inconclusive

Nowak-Wegrzyn et al. (17), Sicherer and Sampson (18), Cianferoni (19), Tordesillas et al. (20), and Morita et al. (21).

#### 3.2.2 Treatment and follow-up

The primary treatment for WA is strict elimination of wheat and wheat-derived products from the diet. In contrast to celiac disease, exclusion of other gluten-containing grains such as barley or rye is generally unnecessary unless cross-reactivity has been demonstrated (18).

Patient education plays a central role and should focus on label reading, early recognition of allergic symptoms, and, when indicated, the availability of rescue medication such as epinephrine auto-injectors. In pediatric patients, regular follow-up visits and laboratory testing are advised to monitor for potential resolution of the allergy over time (19).

Although allergen-specific immunotherapy is being explored as a therapeutic option, it is not yet established as standard practice (21).

### 3.3 Non-celiac gluten sensitivity

NCGS is defined by the onset of gastrointestinal and extra-intestinal symptoms following gluten ingestion in individuals without evidence of CD or WA. Symptoms usually improve upon gluten withdrawal and recur after reintroduction. Unlike CD and WA, no validated biomarkers exist for NCGS, making diagnosis difficult and largely dependent on clinical assessment and exclusion of alternative conditions (23, 24).

Typical gastrointestinal complaints include abdominal pain, bloating, diarrhea, and constipation, whereas extra-intestinal manifestations may involve fatigue, headache, musculoskeletal pain, and cognitive disturbances such as “brain fog” (23, 25).

The underlying mechanisms of NCGS are not fully understood. Current evidence points toward a predominant role of innate immunity, rather than adaptive responses (23, 26). Amylase–trypsin inhibitors (ATIs), naturally occurring wheat proteins, have been implicated in activating toll-like receptor 4 (TLR4), thereby promoting intestinal inflammation (23). Additionally, fermentable oligo-, di-, monosaccharides, and polyols (FODMAPs)—particularly fructans found in wheat—may contribute to gastrointestinal symptoms, although they are unlikely to explain the extra-intestinal features commonly reported in NCGS (27, 28).

#### 3.3.1 Clinical manifestations and diagnosis

The diagnostic process for NCGS is based on systematically excluding CD and WA. This typically involves serological testing for CD-specific antibodies and skin prick tests (SPT) or serum-specific IgE to rule out WA. Once these conditions are excluded, improvement of symptoms on a gluten-free diet and their recurrence following gluten reintroduction may support the diagnosis.

According to the Salerno Experts' Criteria, a double-blind, placebo-controlled crossover challenge represents the gold standard for diagnosis. However, given its complexity, this approach is rarely feasible in everyday clinical practice (23). In routine settings, clinicians often rely on the pattern of symptom remission after gluten withdrawal and recurrence after re-exposure (24).

An important aspect to consider in the clinical evaluation of NCGS is its substantial overlap with irritable bowel syndrome (IBS) (9). Both conditions share common gastrointestinal manifestations, including abdominal pain, bloating, altered bowel habits, and variable symptom fluctuation, which can complicate differential diagnosis. Recent studies have suggested that a subset of patients with IBS, particularly those with diarrhea-predominant or mixed subtypes, may experience symptom improvement on gluten-free or low-FODMAP diets, highlighting potential shared pathophysiological mechanisms (29–31). These mechanisms may include visceral hypersensitivity, altered intestinal permeability, low-grade mucosal inflammation, and activation of the innate immune system. Moreover, wheat components other than gluten—such as amylase–trypsin inhibitors and fructans—can act as triggers in both NCGS and IBS, further blurring the clinical boundaries between the two entities (9, 29). Careful diagnostic assessment is therefore required to distinguish NCGS from IBS, ensuring that dietary interventions are tailored appropriately and unnecessary dietary restrictions are avoided.

#### 3.3.2 Treatment and follow-up

A GFD remains the primary therapeutic approach for NCGS. In contrast to CD, the required level of dietary strictness and the long-term duration of gluten avoidance are not well-defined, and some individuals appear to tolerate small amounts of gluten without symptoms (25).

Regular follow-up is recommended to monitor adherence, evaluate nutritional status, and track symptom progression (5, 23). Importantly, unnecessary adoption of a GFD in individuals without medical indications carries the risk of nutritional deficiencies and should therefore be pursued with caution (5).

### 3.4 Gluten-free diet: beyond treatment

#### 3.4.1 Nutritional risks of GFD

Although a GFD is indispensable for patients with CD and frequently adopted by those with NCGS, it may present nutritional challenges, particularly when followed without medical necessity. The increasing popularity of GFD among the general population has intensified concerns about its long-term health implications (32, 33).

Several studies indicate that processed gluten-free foods often contain lower amounts of protein and fiber while providing higher levels of saturated fat and sugar compared to gluten-containing alternatives (34, 35). Moreover, the lack of systematic micronutrient fortification in gluten-free products can predispose individuals to deficiencies (36, 37).

##### 3.4.1.1 Micronutrient deficiencies

Nutritional inadequacies on a GFD are common when the diet is poorly balanced. Key deficiencies include:

- Iron: Frequently deficient in CD both at diagnosis and follow-up. The lack of iron fortification in gluten-free foods and the limited bioavailability of non-heme iron contribute to persistent anemia (38, 39).
- Folate and B vitamins: Wheat-based foods are major contributors of folate, thiamine, niacin, and riboflavin, particularly in regions with mandatory fortification. Gluten-free products often fail to compensate, increasing cardiovascular risk through elevated homocysteine (40).
- Vitamin B12: Deficiency may occur due to malabsorption or inadequate intake, especially in elderly individuals or those with neurological symptoms (39).
- Vitamin D and calcium: Essential for bone health but often insufficient in GFDs, increasing risk for osteopenia and osteoporosis (38, 41, 42).
- Magnesium and zinc: Reduced intake of whole grains, legumes, and nuts leads to deficiencies that impair enzymatic activity, immunity, and metabolism (39, 43).
- Fiber: GFDs are consistently low in fiber, contributing to constipation, reduced satiety, and unfavorable microbiota changes (34, 44).
- Selenium and Copper: Less frequently studied, but deficiencies can compromise antioxidant defense, thyroid function, and cardiovascular health (39).
- Vitamin K: In cases of fat malabsorption, deficiency may affect coagulation and bone metabolism (38).

To counteract these risks, patients should prioritize naturally gluten-free, nutrient-rich foods such as pseudo-cereals (e.g., quinoa, buckwheat, amaranth), legumes, fruits, and vegetables, and incorporate fortified gluten-free products when available. Ongoing dietary supervision by trained dietitians is strongly recommended (36).

##### 3.4.1.2 Macronutrient imbalances and food quality

Processed gluten-free foods are often nutritionally inferior. They may contain:

- Excess saturated fats and refined sugars added to improve palatability.
- Lower-quality protein, as common gluten-free flours (rice, corn) lack essential amino acids.
- Reduced fiber, negatively impacting satiety, gut function, and metabolic health.
- A higher glycemic index, which can impair glycemic control in individuals with metabolic disorders (34, 44).

##### 3.4.1.3 Gut microbiota and functional implications

Evidence suggests that long-term GFD may induce dysbiosis, characterized by decreased levels of beneficial bacteria such as *Bifidobacteria* and *Lactobacilli* and increased prevalence of potentially harmful taxa like *Enterobacteriaceae* (45). These shifts, largely attributable to reduced intake of whole-grain fibers, may compromise immune regulation, barrier integrity, and nutrient metabolism, particularly in those without a medical indication for GFD.

##### 3.4.1.4 Special considerations for non-celiac individuals

Among individuals without CD or NCGS, adherence to a GFD for perceived health benefits may reduce overall dietary quality. Studies show reduced intake of essential nutrients alongside increased consumption of processed gluten-free snacks, often rich in sugars and fats (33, 36). Without professional guidance, this population may inadvertently heighten their risk of chronic disease.

#### 3.4.2 GFD and weight change

Weight outcomes on a GFD can be bidirectional. In newly diagnosed CD patients, improved nutrient absorption after mucosal healing may result in weight gain, which can reduce dietary adherence in some cases (46). Conversely, an increasing number of patients are overweight or obese at diagnosis, raising questions about GFD's metabolic impact (47, 48).

A Finnish nationwide study (*n* = 698) reported BMI improvements across diagnostic categories after 1 year of GFD, with underweight patients gaining weight and overweight/obese patients showing partial normalization (47). Brambilla et al. observed BMI stabilization in children after diagnosis, with fewer underweight cases and only a slight increase in overweight prevalence (49). A 2022 prospective study (*n* = 215) found significant weight gain in patients following GFD for more than 2 years (50). A 2023 systematic review (*n* = 45 studies, ~28,000 participants) showed BMI redistribution, with some individuals moving into higher or lower BMI categories, but no increased risk of pathological weight gain (51). Another meta-analysis confirmed increased weight and fat mass in CD patients but no significant changes in healthy controls (52).

Overall, these findings suggest that GFD may normalize weight in CD patients, but long-term risks related to processed gluten-free foods (high in fat and salt) remain a concern (33).

#### 3.4.3 GFD and eating disorders

Strict dietary control may predispose certain individuals to maladaptive eating behaviors. Shared features between CD and eating disorders (EDs)—including weight loss, fatigue, and nonspecific gastrointestinal complaints—can complicate diagnosis (53, 54).

The constant vigilance required to maintain a GFD may mimic or exacerbate disordered eating, particularly in adolescents and women (55, 56). A 2021 meta-analysis reported a pooled prevalence of EDs of 8.88% among CD patients, markedly higher than the general population (57).

Strong associations have been reported between CD and anorexia nervosa (AN). A Swedish cohort study (*n* = 107,000) found increased hazard ratios for AN following CD diagnosis and vice versa (58, 59). Smaller surveys confirm changes in eating attitudes post-diagnosis, with heightened awareness and negative emotions toward food (60).

Clinicians should remain vigilant for ED symptoms in CD patients, as they can compromise adherence to GFD and adversely affect psychological and physical health (61).

#### 3.4.4 GFD and cardiovascular risk

While GFD is the only effective treatment for CD (62), it is not inherently cardioprotective. Gluten-free starches and flours typically have higher glycemic indices and lower fiber and nutrient density (63). Adequate fiber intake is essential for cardiovascular health due to its lipid-lowering and anti-inflammatory properties (64, 65).

Evidence regarding the cardiometabolic effects of GFD is mixed. Some studies and reviews report improvements in HDL cholesterol, systolic blood pressure, and inflammatory markers, but increases in total and LDL cholesterol (66–72). Pediatric cohorts have shown increases in both total and HDL cholesterol after adopting GFD (71). Differences in lipid responses between genders have also been observed (73). Lipoprotein(a), an independent cardiovascular risk factor, appears unaffected by GFD (74–76).

Overall, GFD may contribute to metabolic alterations, including weight gain, dyslipidemia, and glucose imbalance, particularly when based on processed gluten-free foods (77, 78). Patients should be counseled on balanced dietary choices to mitigate these risks.

#### 3.4.5 Environmental factors in CD

Although genetic predisposition (HLA-DQ2/DQ8) is central to CD development, environmental influences also play a role. Gluten exposure is the most critical factor, but breastfeeding, early-life infections, microbiome alterations, and antibiotic use may modulate risk (79–81).

Early observational studies suggested protective effects of breastfeeding and timing of gluten introduction, but large prospective trials such as PreventCD and TEDDY found no significant influence on CD risk (82, 83). As a result, ESPGHAN guidelines do not recommend breastfeeding as a preventive measure against CD, although it is still encouraged for its many other health benefits (84).

Additional concerns have been raised regarding industrial food processing. Use of microbial transglutaminase as an additive may negatively affect gluten-sensitive individuals, potentially influencing disease risk (85).

## 4 Discussion

Gluten-related disorders comprise a heterogeneous spectrum of conditions—CD, WA, and NCGS—that present with overlapping symptoms but differ substantially in immunological mechanisms, diagnostic criteria, and management. Despite notable progress in diagnostic strategies, differentiating between these conditions remains clinically challenging, particularly in the absence of reliable biomarkers for NCGS and the wide variability of gastrointestinal and extra-intestinal manifestations. These diagnostic uncertainties can contribute both to delayed treatment initiation and to unnecessary adoption of dietary restrictions.

The GFD represents the cornerstone of therapy for CD and a valuable approach for many cases of WA and NCGS. Nevertheless, its increasing popularity among individuals without confirmed diagnoses has raised legitimate concerns. Evidence consistently shows that GFD, especially when based on industrially processed gluten-free products, is associated with micronutrient deficiencies (iron, folate, B vitamins, vitamin D, calcium, zinc, magnesium), reduced dietary fiber, and potential alterations in gut microbiota. Moreover, imbalances in macronutrient composition, higher glycemic index, and increased levels of fats and sugars in commercial gluten-free products may predispose individuals to metabolic complications, including overweight, obesity, and cardiovascular risk.

These issues are particularly relevant in pediatric populations. Unlike adults, children require carefully tailored diets to support optimal growth, bone mineralization, and neurodevelopment. Applying restrictive dietary interventions without medical justification may compromise nutritional adequacy and expose younger patients to psychosocial burdens, including heightened risk of maladaptive eating behaviors. Such considerations highlight the duality of GFD: indispensable for those with CD and often beneficial for WA and NCGS, yet potentially harmful when adopted indiscriminately.

A key strength of this narrative review lies in its comprehensive scope, integrating recent findings across multiple domains—including pathogenesis, diagnosis, management, and nutritional outcomes—while drawing on a wide range of clinical studies and systematic reviews. This broad perspective allows for an up-to-date synthesis of the clinical, nutritional, and public health implications of gluten-related disorders. However, certain limitations must be acknowledged. As a narrative review, this work is not based on systematic evidence appraisal, and the selection of studies may have introduced bias. Furthermore, the heterogeneity of available studies, varying diagnostic definitions of NCGS, and differences in study populations and dietary assessments complicate direct comparison of findings. Finally, the rapidly evolving nature of research in this field means that new data may soon refine or challenge current conclusions.

Taken together, these strengths and limitations underscore the importance of interpreting the present findings as a broad overview rather than definitive guidance, while pointing to the need for well-designed prospective studies to clarify unresolved questions.

While the present review supports the therapeutic value of the GFD for patients with confirmed CD, WA, and NCGS, it is important to acknowledge the ongoing debate regarding the extent of gluten's role in symptom generation, particularly in NCGS and IBS. Some studies suggest that gluten itself is not the primary trigger in many self-reported NCGS cases, pointing instead to other wheat components such as fructans or amylase–trypsin inhibitors as symptom-inducing agents (27, 28). Conversely, other investigations have demonstrated reproducible symptom recurrence after blinded gluten challenges, supporting gluten as a distinct trigger in a subset of sensitive individuals (23, 25). Similarly, while numerous reports highlight nutritional deficiencies and cardiometabolic concerns associated with GFD, some evidence indicates improved metabolic profiles and quality of life when the diet is appropriately balanced and supervised (70). These contrasting perspectives emphasize that the impact of gluten and GFD is heterogeneous and context-dependent. Future studies should therefore aim to stratify patients according to immunological and metabolic phenotypes, enabling more precise dietary recommendations and minimizing unnecessary restrictions.

## 5 Conclusions

The growing recognition of gluten-related disorders has improved the diagnosis of CD and expanded clinical awareness of WA and NCGS. At the same time, the widespread self-prescribed adoption of the GFD in individuals without medical necessity highlights the need for caution. While GFD is indispensable for CD and often beneficial in WA and NCGS, its indiscriminate use can result in micronutrient deficiencies, altered gut microbiota, and potential cardiometabolic risks, particularly when the diet relies on processed gluten-free products.

This narrative review emphasizes the importance of accurate differential diagnosis, individualized dietary counseling, and continuous follow-up in both adult and pediatric populations. Particular attention must be paid to children, where nutritional adequacy and healthy growth should remain the primary objectives. Future research should prioritize high-quality prospective studies to better define the long-term nutritional and metabolic consequences of GFD, with special focus on pediatric populations and individuals without confirmed diagnoses. Such evidence is essential to balance the therapeutic benefits of GFD with its potential risks, ensuring safe, effective, and patient-centered care.
